# Targeting extracellular vesicles to injured tissue using membrane cloaking and surface display

**DOI:** 10.1186/s12951-018-0388-4

**Published:** 2018-08-30

**Authors:** Travis J. Antes, Ryan C. Middleton, Kristin M. Luther, Takeshi Ijichi, Kiel A. Peck, Weixin Jane Liu, Jackie Valle, Antonio K. Echavez, Eduardo Marbán

**Affiliations:** Smidt Heart Institute, Cedars-Sinai Medical Center, 8700 Beverly Blvd., Davis Building, Los Angeles, CA 90048 USA

**Keywords:** Extracellular vesicles, EV, Exosome, Phospholipid, Membrane anchor, Streptavidin, Biotin, Homing peptides, Targeting antibodies, Qdots, Myoblasts, Cardiomyocytes, Ischemia/reperfusion injury, Infarction, Biodistribution, NanoSight NTA, Surface display, Lactadherin, C1C2 domain fusions

## Abstract

**Background:**

Extracellular vesicles (EVs) and exosomes are nano-sized, membrane-bound vesicles shed by most eukaryotic cells studied to date. EVs play key signaling roles in cellular development, cancer metastasis, immune modulation and tissue regeneration. Attempts to modify exosomes to increase their targeting efficiency to specific tissue types are still in their infancy. Here we describe an EV membrane anchoring platform termed “cloaking” to directly embed tissue-specific antibodies or homing peptides on EV membrane surfaces ex vivo for enhanced vesicle uptake in cells of interest. The cloaking system consists of three components: DMPE phospholipid membrane anchor, polyethylene glycol spacer and a conjugated streptavidin platform molecule, to which any biotinylated molecule can be coupled for EV decoration.

**Results:**

We demonstrate the utility of membrane surface engineering and biodistribution tracking with this technology along with targeting EVs for enhanced uptake in cardiac fibroblasts, myoblasts and ischemic myocardium using combinations of fluorescent tags, tissue-targeting antibodies and homing peptide surface cloaks. We compare cloaking to a complementary approach, surface display, in which parental cells are engineered to secrete EVs with fusion surface targeting proteins.

**Conclusions:**

EV targeting can be enhanced both by cloaking and by surface display; the former entails chemical modification of preformed EVs, while the latter requires genetic modification of the parent cells. Reduction to practice of the cloaking approach, using several different EV surface modifications to target distinct cells and tissues, supports the notion of cloaking as a platform technology.

**Electronic supplementary material:**

The online version of this article (10.1186/s12951-018-0388-4) contains supplementary material, which is available to authorized users.

## Background

### Extracellular vesicles and exosomes

Cells secrete extracellular vesicles (EVs) with a broad range of diameters and functions, including apoptotic bodies (1–5 μm [1]), microvesicles (100–1000 nm in size [2]), and vesicles of endosomal origin, known as exosomes (50–150 nm [3, 4]). Exosomes express characteristic surface tetraspanin proteins, such as CD9, CD63 and CD81 [5]. Internal RNA cargoes are an additional feature of EVs; notably, small ncRNA, circular RNA, miRNA, mRNA, tRNA and lncRNA are commonly detected in exosome preparations [6]. Exosomes function as shuttles with intercellular signaling capabilities. These EVs promote cancer metastasis [7, 8], play crucial roles in embryonic development [9, 10], modulate immune responses [11, 12], accelerate soft tissue wound healing [13, 14], improve skeletal myopathy in Duchenne muscular dystrophy (DMD) models [15], and support heart repair after myocardial infarction (MI) [16, 17]. Here, we refer to the collective extracellular fraction of vesicles, including exosomes, as EVs, and routinely characterize our EV preparations for vesicle size, concentration, RNA content and surface protein phenotype. Due to their potential as therapeutic candidates for numerous applications, we sought to engineer EVs with enhanced accumulation and uptake in selected target tissues.

### Cardiosphere-derived cells (CDCs) and EVs (CDC-EV)

Here we used EVs made from CDCs, which were originally described in 2007 as a distinct cardiac progenitor cell population generated in primary culture from human heart samples [18]. CDCs are of intrinsic cardiac origin, multipotent and clonogenic. A number of clinical trials have used or are using CDCs [19]. The initial rationale was that CDCs would work canonically, i.e., engraft, proliferate and differentiate into new myocardium. However, preclinical studies have revealed that, despite being progenitor cells, CDCs do not work that way. Few (≪ 1% of injected) cells are measurable 3–4 weeks after transplantation, but functional and structural benefits persist for at least 6 months post-treatment [20]. During the ~ 2 weeks that appreciable numbers of transplanted CDCs persist in the tissue, CDCs indirectly induce cardiomyogenesis in the host myocardium [21]. Many, if not most, of the effects of EVs are seemingly mediated by their RNA contents, specifically miRs and other noncoding RNAs (ncRNAs) [17, 22]. CDC-EV contain diverse RNA (e.g., miR-146a [16], miR-181b [23], Y RNA fragments [24]) and protein [16, 23, 25] cargo. CDC-EV have the capacity to reduce MI-related scar formation [16, 23], increase cardiomyocyte proliferation [16], reduce fibrosis [25], support cardioprotection [26, 27], and modulate inflammation [23]. Potentially superior features over the parent stem cell therapies include drug product stability, immune tolerability, and systemic efficacy. However, the biodistribution of wild type EVs may or may not be favorable for a desired application. To enhanced EV development for therapeutics, what’s needed is a way to program specific tissue localization such that EVs accumulate in the desired target tissue after non-localized delivery, e.g. by simple intravenous (IV) injection.

### Current methods for engineering EV surfaces

Attempts to modify EVs to increase their targeting efficiency are not new [28], but are still in their infancy. The first technique to demonstrate successful engineering of a parent cell line to generate altered EVs is using a technology termed “surface display” [29]. This is done by cloning the protein sequence to be “displayed” on the vesicle membrane surface as a translational fusion to the C1C2 domain of the human lactadherin protein. The C1C2 domain is placed at the C-terminus of the fused sequence and, when expressed in parent cells, this signal will traffic the entire protein into secreted EVs and position the N-terminal region outward on the EV surface. This approach has shown success in displaying carcinoembryonic antigen (CEA) and HER2 on EVs for enhancing vaccine development [30] and recently to target HER2^+^ breast cancer cells with EVs displaying scvHER2 antibodies to deliver mRNA prodrugs for anti-cancer activity [31]. Such C1C2 fusion proteins demonstrated significant immune response improvements of therapeutic anti-tumor effects in HER2^+^ transgenic animal models. Other methods of genetically modifying parent cells for to produce “pseudotyped” EVs have been described [32, 33]. EV pseudotyping employs viral protein sequences to translocate protein fusions to the membrane surfaces of EVs for tissue targeting. An archetypal example of this approach utilized the central nervous system–specific rabies viral glycoprotein (RVG) peptide, that specifically binds to the acetylcholine receptor, to target EVs to the central nervous system [33]. For proof-of-concept, these RVG-pseudotyped EVs were also loaded with siRNAs to knockdown BACE1, a key factor in Alzheimer’s disease pathogenesis [34, 35]. This study demonstrated that RVG-engineered EVs achieved ~ 40% knockdown of BACE1 in vivo [33]. A second approach involves the ex vivo “fusing” of EVs isolated from genetically-engineered cells with particular surface proteins embedded in synthetic liposomes [36]. These hybrid micelle:EVs are created through multiple rounds of freeze–thaw mixing, and have an altered lipid composition due to addition of exogenous lipid micelles. The cellular uptake of EV–liposome hybrids led to only a modest improvement over native EVs alone [36].

In this report, we describe a membrane engineering methodology to directly embed EV surfaces ex vivo with an anchor conjugated to streptavidin. This provides a modular platform where any biotinylated molecule, such as a fluorescent molecule for tracking biodistribution, can be combined with tissue-targeted antibodies or homing peptides to facilitate engineered EV uptake in cells of interest. The targeting approach, which we have termed “cloaking”, involves adding modified glycerol-phospholipid-PEG conjugates (DMPE-PEG) to isolated EVs in solution. DMPE-PEG embeds into vesicle lipid bilayer membranes and serves as an anchor for coupling biotinylated fluorescent molecules or ligand proteins [37, 38]. We demonstrate cell uptake assays and whole animal biodistribution studies using biotinylated fluorophores cloaked on EVs. This tagging method was then extended to show utility in directing enhanced EV uptake in cells and tissues of interest with combinations of biotinylated targeting antibodies and tissue homing peptides. Finally, we compare the cloaking approach with the better-established surface display approach, using the identical targeting peptide in the same model of myocardial injury.

## Results

### Cloaking EVs with fluorescent molecules

**Fig. 1:**
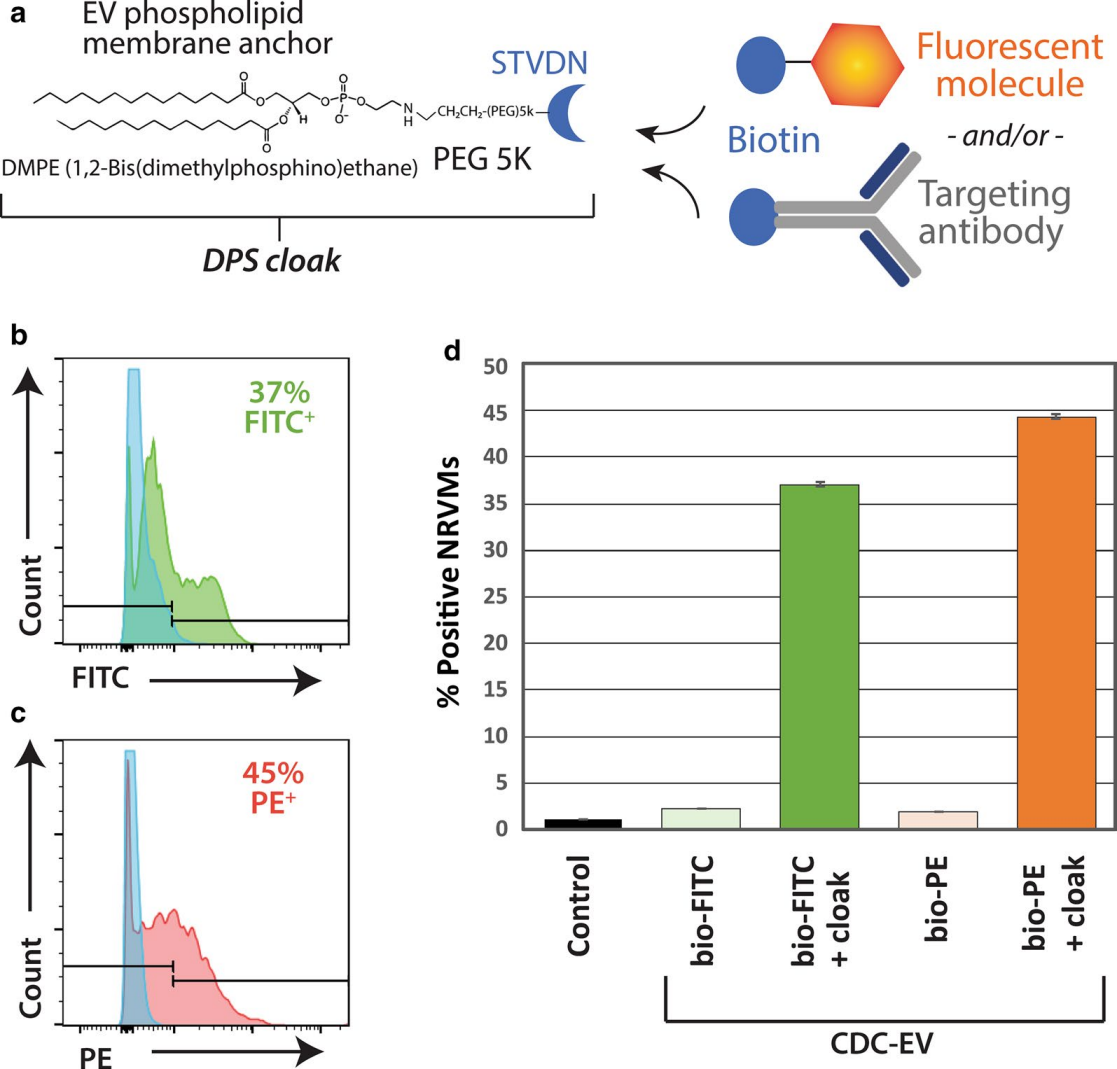
Exosome fluorescent cloaking. **a** Schematic of cloaking technology depicting the three components: DMPE phospholipid membrane anchor, Polyethylene glycol (PEG) 5K spacer and streptavidin platform molecule (STVDN), together abbreviated DPS. To the right in **a**, example types of biotinylated molecules that can be coupled to the DPS membrane platform are shown. Representative FACS plots depicting NRVM uptake of CDC-EV cloaked with bio-FITC (**b**) or bio-PE (**c**), gates are indicated. **d** Pooled data from **b**, **c**. n = 4 wells per experimental group

We conjugated streptavidin (STVDN) with DMPE-PEG to create a modular EV membrane anchoring platform (DMPE-PEG-STVDN; DPS). Thus, any biotinylated molecule (e.g., antibodies) can be coupled to the DPS anchor to decorate vesicle membranes for targeted delivery. A schematic of the membrane cloak anchoring strategy is depicted in Fig. 1a. To demonstrate the feasibility of this technology, CDC-EV were isolated, cloaked with DPS, then coupled with a biotinylated fluorescent marker bio-FITC or bio-PE. In this report, we utilize ultrafiltration techniques [39, 40] to enrich and concentrate EVs from CDC conditioned media. The CDC-EV were incubated with the cloaks for 10 min, then after an ultrafiltration step to remove unincorporated cloaks, the bio-FITC and bio-PE cloaked CDC-EV were added to neonatal rat ventricular myocytes (NRVMs) in culture. The assays were allowed to proceed for 4 h, then the NRVMs were subjected to FACS analysis to quantitate fluorescent CDC-EV uptake. The results shown in Fig. 1. Indicate rapid uptake of the cloaked bio-FITC CDC-EV (Fig. 1b) and bio-PE CDC-EV (Fig. 1c) when compared to unlabeled CDC-EV with approximate equal uptake rate of about 40% neonatal rat ventricular myocytes (NRVMs) with fluorescent intensities well over background (Fig. 1c).

### Targeting cardiac fibroblasts with antibody cloaks

**Fig. 2:**
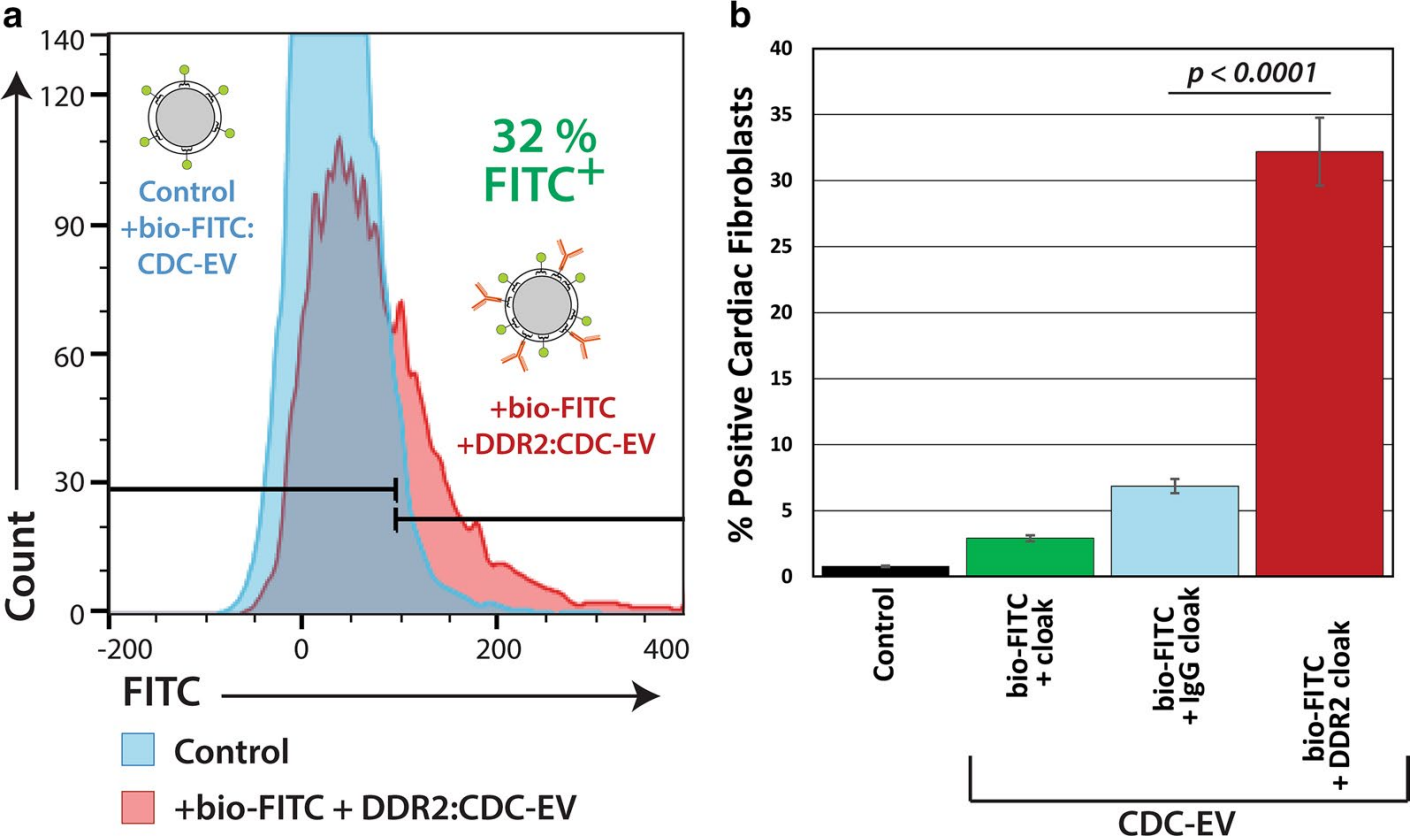
CDC-EV DDR2 cloak differential uptake in cardiac fibroblasts. **a** Representative FACS histograms of rat cardiac fibroblast uptake assays of CDC-EV with targeting antibody cloaks. Graphic inset: CDC-EV diagrams showing the cloaks added. **b** Graphical analysis of pooled data from (**a**) of CDC-EV uptake levels in rat cardiac fibroblasts in culture. n = 3 wells per experimental group

The process of embedding membrane anchors does not deform the CDC-EV (Additional file 1: Figure S1) and apparently does not abrogate cellular uptake in cardiomyocytes as seen in Fig. 1. Cardiomyocytes comprise a small portion of the cells in healthy heart tissue [41]; many of the remaining cells are cardiac fibroblasts [42]. These cells play an active role in the development of hypertrophy [43] and dysfunctional cardiomyocyte remodeling [44–46]. The mechanisms of cardiomyocyte–fibroblast communication are poorly understood. Our preliminary work with rat cardiac fibroblasts has revealed that these cells are highly resistant to CDC-EV uptake. In contrast, prior work with dermal fibroblasts has shown that CDC-EV are readily taken up and confer profound salutary changes in fibroblast phenotype. Thus, developing methods to successfully target CDC-EV to cardiac fibroblasts may prove therapeutically-relevant. We chose to target Discoidin Domain Receptor tyrosine kinase 2 (DDR2), an abundant cardiac fibroblast surface marker [47]. Similar to the methodology to examine uptake with macrophages, we cloaked CDC-EV with bio-DDR2 and bio-FITC (bio-DDR2/FITC) or bio-IgG/FITC (as non-targeting control). Neonatal rat cardiac fibroblasts were isolated as described [48] and exposed to CDC-EV (bio-DDR2/FITC or bio-IgG/FITC). Twelve hours later, cells were harvested and analyzed for CDC-EV uptake by flow cytometry. Cardiac fibroblasts treated with bio-DDR2/FITC, in contrast to bio-IgG/FITC, CDC-EV revealed significantly greater uptake (30% vs. ~ 5%, p < 0.0001) (Fig. 2a, b).

### Utilizing tissue homing peptides as cloaks to target CDC-EV

**Fig. 3:**
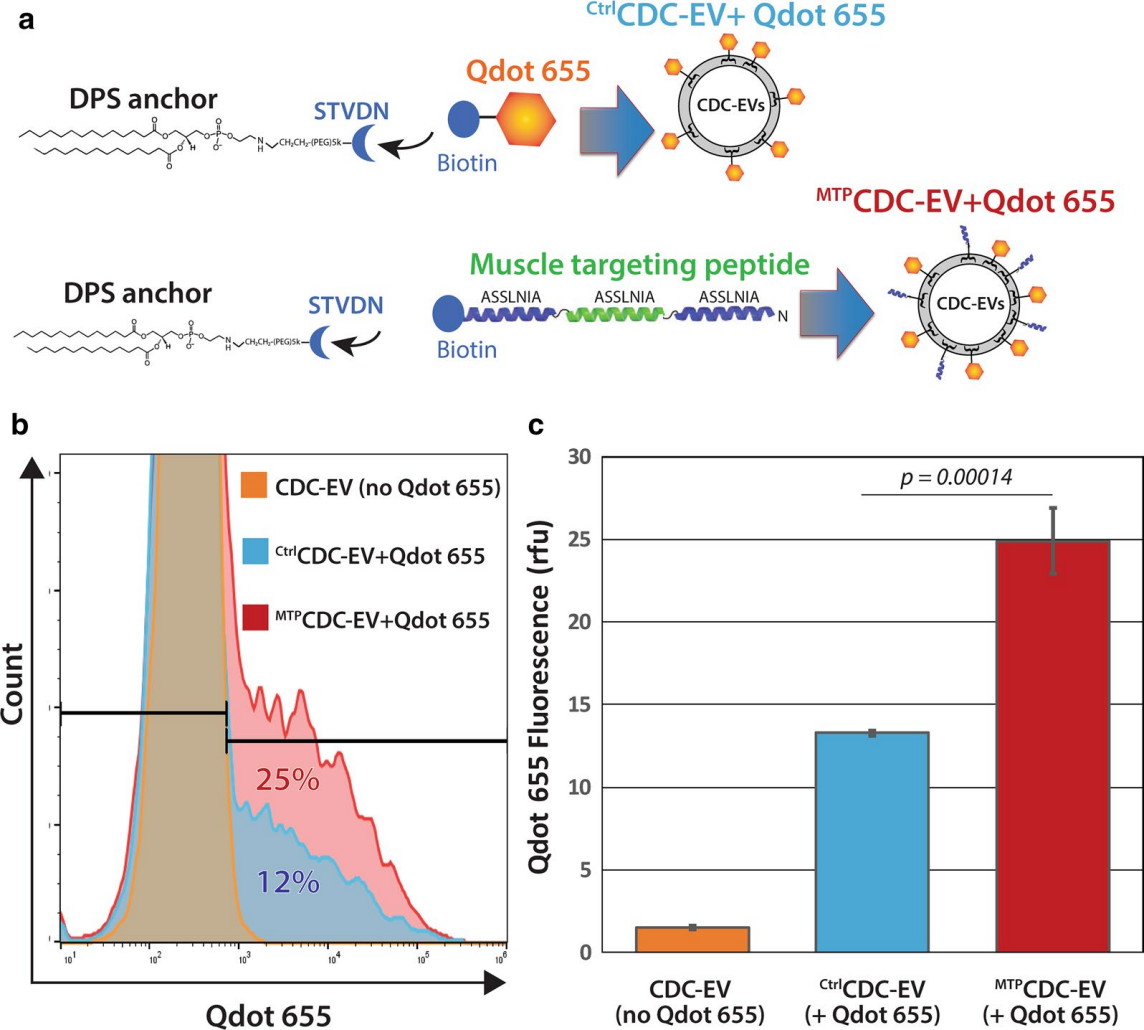
Uptake assays of CDC-EV with Qdot 655 and muscle targeting peptide cloaks. **a** Schematic of the design of MTP and Qdot 655 membrane cloaks. **b** Representative FACS histograms of mouse H2K mdx myoblast uptake assays of CDC-EV with muscle targeting peptide (MTP) and Qdot 655 labeling cloaks versus controls. **c** Graphical analysis of pooled data from (**b**) of CDC-EV uptake levels. n = 3 wells per experimental group

Phage display in vitro and in vivo screens have identified several unique, short peptide sequences that confer tissue homing specificities [49, 50]. Homing peptides targeting tissues such as lung [50, 51], brain [52, 53], kidney [54], muscle [55, 56], and ischemic myocardium [57] have been reported. CDC-EV may potentially have clinical applications for treating muscular dystrophies in DMD [15]. Thus, we selected the muscle homing peptide sequence ASSLNIA [56] to assess if cloaking CDC-EV can confer enhanced muscle cell uptake. A homing peptide molecule bearing three copies of the peptide sequence ASSLNIA, separated by two glycine residues in between, was synthesized along with a biotin group conjugated to the C-terminus. The muscle targeting peptide (termed MTP) was used in combination with bio-Qdot 655 fluorescent molecules for tracking uptake with mouse H2K *mdx* myoblasts [58, 59] in vitro. A schematic of the cloaking molecules used in these studies is shown in Fig. 3a. The dual-cloaked CDC-EV were again analyzed for vesicle size, concentration and fluorescent tagging using dynamic light scattering in visible or fluorescent modes by NanoSight [4] methods to visualize and quantitate Qdot 655-labeled EVs (Additional file 2: Figure S2). Equal amounts of Qdot 655-labeled, control or MTP-cloaked CDC-EV were incubated with undifferentiated H2K *mdx* myoblasts for 12 h. The cells were then prepared for FACS to quantify levels of cloaked CDC-EV uptake. The FACS histograms shown in Fig. 3b reveal significant enhancement (by nearly 100%) of myoblast uptake (p = 0.00014) of CDC-EV that display MTP cloaks on their surfaces when compared to control EVs (Fig. 3c).

**Fig. 4:**
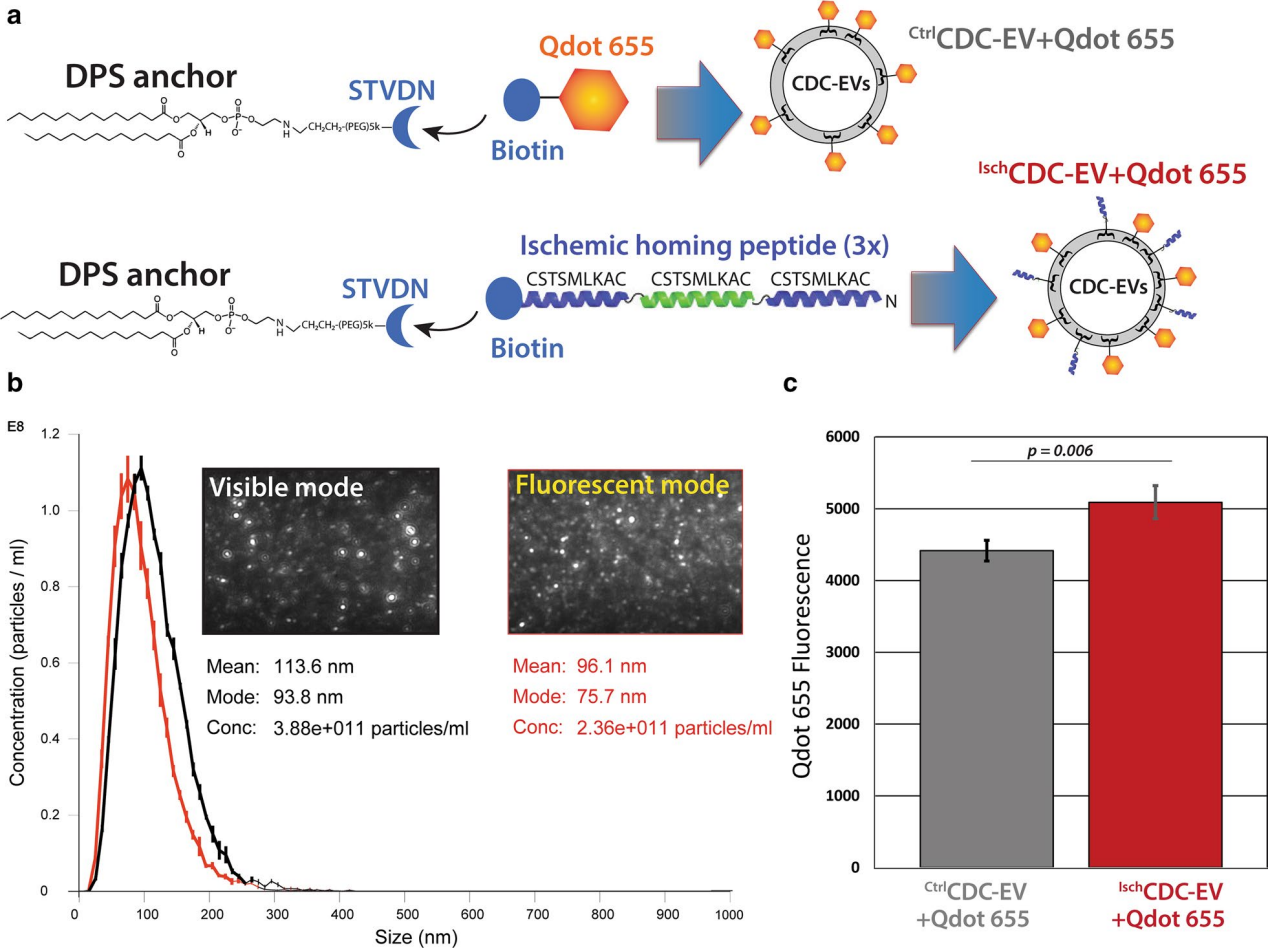
Ischemic NRVM uptake assays of CDC-EV with Qdot 655 and ischemia targeting peptide cloaks. **a** Schematic of the design of Ischemic peptide and Qdot 655 membrane cloaks. **b** Example NanoSight particle tracking profile data for ^Isch^CDC-EV + Qdot 655 in visible and fluorescent modes. **c** FACS graphs of uptake rates of NRVM cells with CDC-EV labeled with Qdot 655−/+ ischemic targeting peptide (Isch) cloaks. Data are plotted as the average of raw Qdot 655 fluorescent readings for nonischemic (Ctrl) versus ischemic (Isch, H_2_O_2_-treated) NRVMs. n = 4 wells per experimental group

Next, we designed another homing peptide cloak to test whether we could program CDC-EV to target injured cells and tissues. An ischemic targeting peptide sequence CSTSMLKAC was initially identified using in vivo phage display screens in mouse ischemia/reperfusion models with damaged myocardial tissue used as the biopanning source [57]. This peptide sequence has also been reported to successfully home synthetic lipid polymeric carrier particles to ischemic myocardium [60] using IV administration of the polyplexes bearing the CSTSMLKAC peptide on their surfaces. Mimicking the design of the muscle targeting peptide cloak, we synthesized an ischemia peptide cloak with three copies of the homing peptide CSTSMLKAC sequence, separated by two glycine spacer residues, and a C-terminal biotin group for coupling to the DPC membrane anchor. The cloaked CDC-EV were analyzed for ischemic peptide and Qdot 655 cloaking using NanoSight NTA methods as before (Fig. 4a) to verify EV recovery after cloaking and assess Qdot 655 labeling efficiency. Rat cardiomyocytes were either cultured untreated or subjected to H_2_O_2_ pre-treatment to model ischemic conditions in vitro as described [26, 61]. Equal particle numbers of ischemic peptide, Qdot 655-cloaked ^Isch^CDC-EV and Qdot 655 control ^Ctrl^CDC-EV were added to NRVMs and allowed to incubate for 12 h. Rates of CDC-EV uptake were quantified for Qdot 655 fluorescence using FACS; data were normalized to non-ischemic NRVM uptake levels for each group and plotted in Fig. 4b. The ischemia targeting peptide cloaks directed greater uptake on NRVMs pre-treated with H_2_O_2_ versus untreated and non-ischemic cloaked CDC-EV by about 13%. The significant enhancement of the ischemia targeting (p = 0.006) via cloaking in vitro led us to pursue this further with rodent infarction models in vivo.

**Fig. 5:**
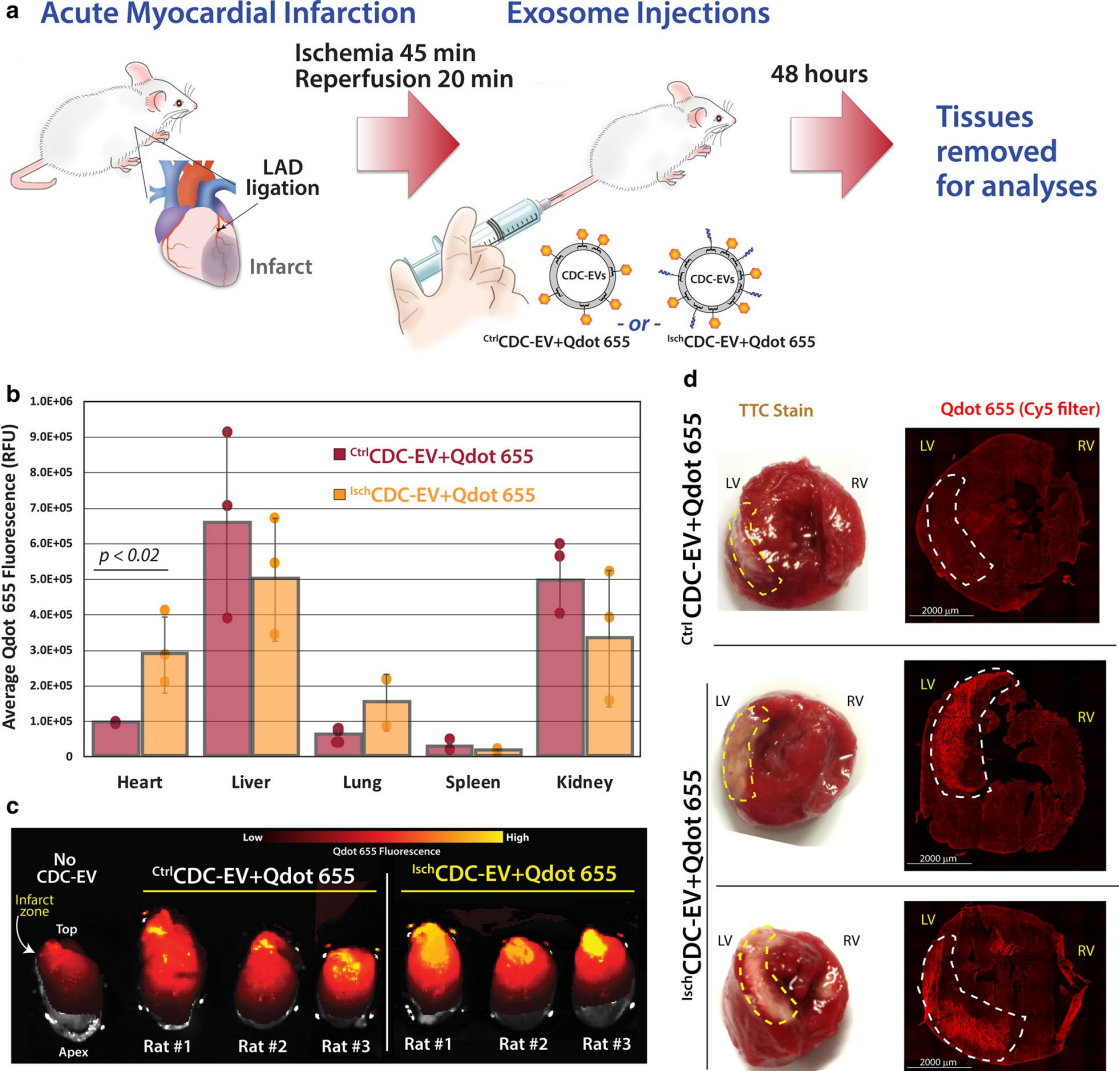
Biodistribution and heart scar localization of targeted CDC-EV. **a** Schematic outlining experimental approach. **b** Graphical representation of whole organ Qdot 655 fluorescent measurements to identify CDC-EV biodistribution in ischemia/reperfusion (I/R) rat study animals. **c** Xenogen whole heart images for Qdot 655 localization of control (Ctrl) and ischemic peptide-targeted (Isch) CDC-EV. **d** Example TTC stains of whole heart slices to identify I/R scar location in rat hearts (blanched regions, left panels) and detailed microscopic fluorescent imaging data (right panels) of slides with thin sections of the same heart tissue to image localization of control ^Ctrl^CDC-EV + Qdot 655 or ischemia-targeted exosomes (^Isch^CDC-EV_+Qdot 655_). Regions of heart scarring due to infarction in TTC stains are outlined in white and corresponding areas in heart tissue sections outlined in yellow. Scar bar indicated at 2000 μm, *LV* left ventricle, *RV* right ventricle. n = 3 rats per sample group, individual rats indicated by a circle

Our in vivo studies with CDC-EV targeting were designed to attain three primary objectives: (1) track CDC-EV major organ biodistribution after simple tail vein injection, (2) assess whether ischemia homing peptide cloaks direct CDC-EV uptake to damaged myocardium and (3) determine whether ischemia is an absolute requirement to attract and enrich ischemia-targeted CDC-EV to heart tissue. The rat ischemia/reperfusion (I/R) model was employed as a model of myocardial infarction coupled to tail vein injections of Qdot 655-tagged ^Ctrl^CDC-EV or Qdot 655-tagged and cloaked with ischemia homing peptides (^Isch^CDC-EV, 10^8^ EVs per animal in 1 mL PBS). The experimental outline is shown in the schematic in Fig. 5a. All test animals underwent transient coronary ligation to induce I/R. Major organs (heart, liver, lung and kidneys) were harvested 48 h after EV injections. Whole organ Qdot 655 fluorescence values were quantitated [62] and plotted in Fig. 5b. As anticipated, the major filtration organs such as liver and kidneys were major locations of EV biodistribution, with a trend towards higher levels of ^Isch^CDC-EV in lungs. EV distribution in whole hearts showed a significantly (p < 0.02) higher level of tracking with ischemia-homing cloaked ^Isch^CDC-EV compared to untargeted ^Ctrl^CDC-EV. This enhanced uptake was further evidenced by whole heart Xenogen fluorescent imaging [63, 64], revealing much higher levels of fluorescence in rats that received ^Isch^CDC-EV injections with highest fluorescence detected in the region that had been subjected to I/R (Fig. 5c). To verify EV distribution to infarcted regions, the hearts were stained with TTC to identify the scar region (blanched white areas [26]), shown in left panels of Fig. 5d. These same hearts were then sectioned, mounted on slides and imaged for Qdot 655 fluorescence. We observed a striking correlation of Qdot 655 fluorescence with the targeted ^Isch^CDC-EV that was not seen with the untargeted ^Ctrl^CDC-EV. Having satisfied our secondary objective to assess whether ischemia homing peptide cloaks could direct CDC-EV to regions of infarcted heart tissue, we next sought to test whether ischemia was an absolute requirement (third experimental objective) for the enhanced biodistribution observed in Fig. 5.

**Fig. 6:**
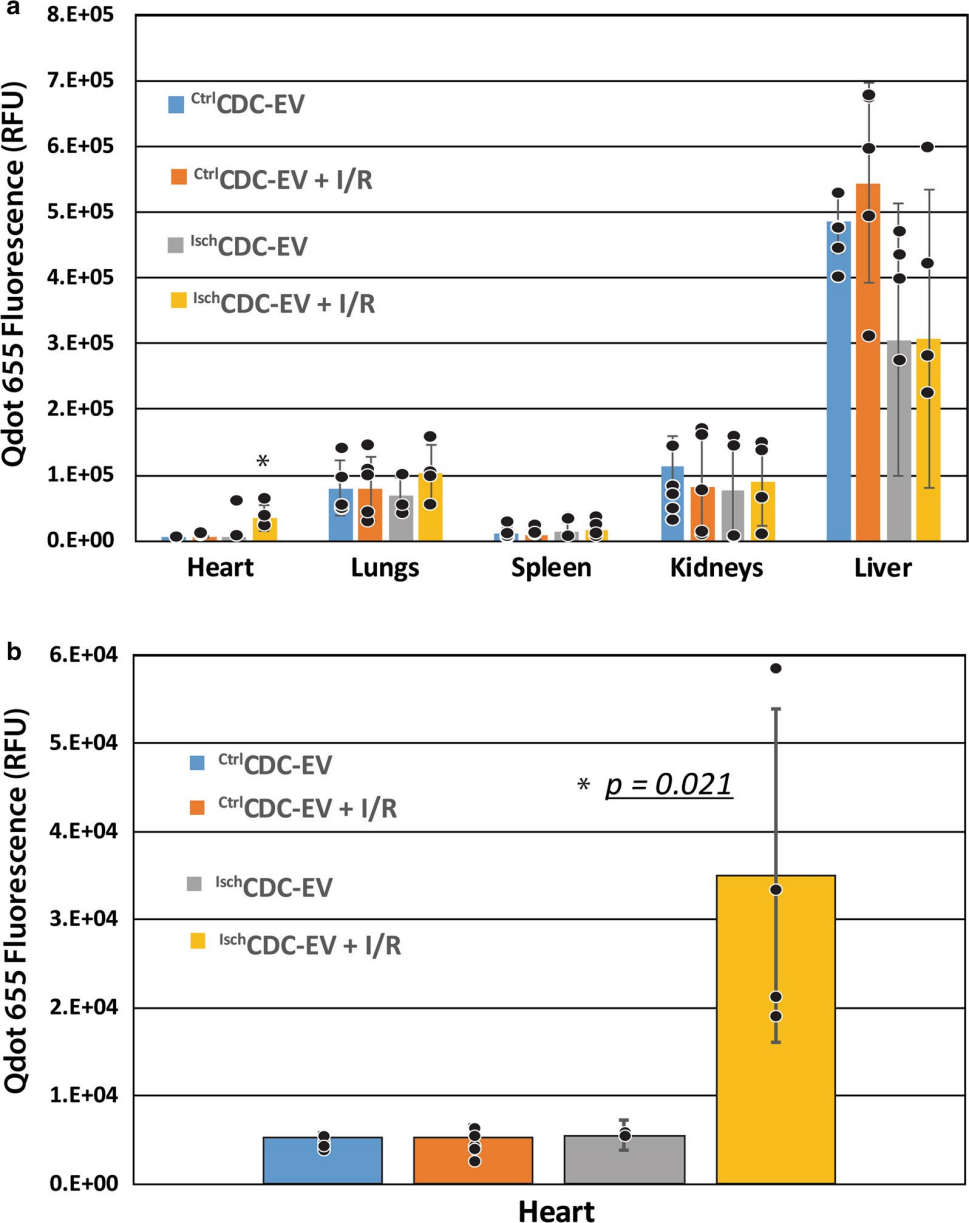
Qdot 655-labeled CDC-EV tissue biodistribution and homing with ischemia. **a** Graphical representation of whole organ Qdot 655 fluorescent measurements to identify Qdot-tagged CDC-EV biodistribution in control versus ischemia/reperfusion (I/R) rat study animals. **b** Bar graph data of untargeted control (^Ctrl^CDC_exo_) or ischemia-targeted ^Isch^CDC-EV homing to heart tissue with or without I/R injury in rat models of myocardial infarction. Y axis represents the raw Qdot 655 fluorescence expressed as relative fluorescence units (RFU). n = 4–5 rats per experimental group, individual rats indicated by a circle

To test this, we utilized the identical rat I/R model outlined in Fig. 5a, except we included an uninfarcted experimental group as a control in the study. Thus, there were four experimental groups total using Qdot 655-tagged EVs, non-targeted ^Ctrl^CDC-EV ± infarction (I/R) and ^Isch^CDC-EV, ± I/R. Again, major organs were harvested 48 h after EV administration for whole tissue Qdot 655 fluorescence quantitation. As observed previously, the primary biodistribution of CDC-EV were to the liver and kidneys, independent of whether I/R was modeled (Fig. 6a). We detected a significant uptake of the targeted ^Isch^CDC-EV in the heart *only* when I/R was applied (Fig. 6b), as compared to the same EVs delivered without I/R injury, and when comparing uptake to ^Ctrl^CDC-EV (p = 0.021).

**Fig. 7:**
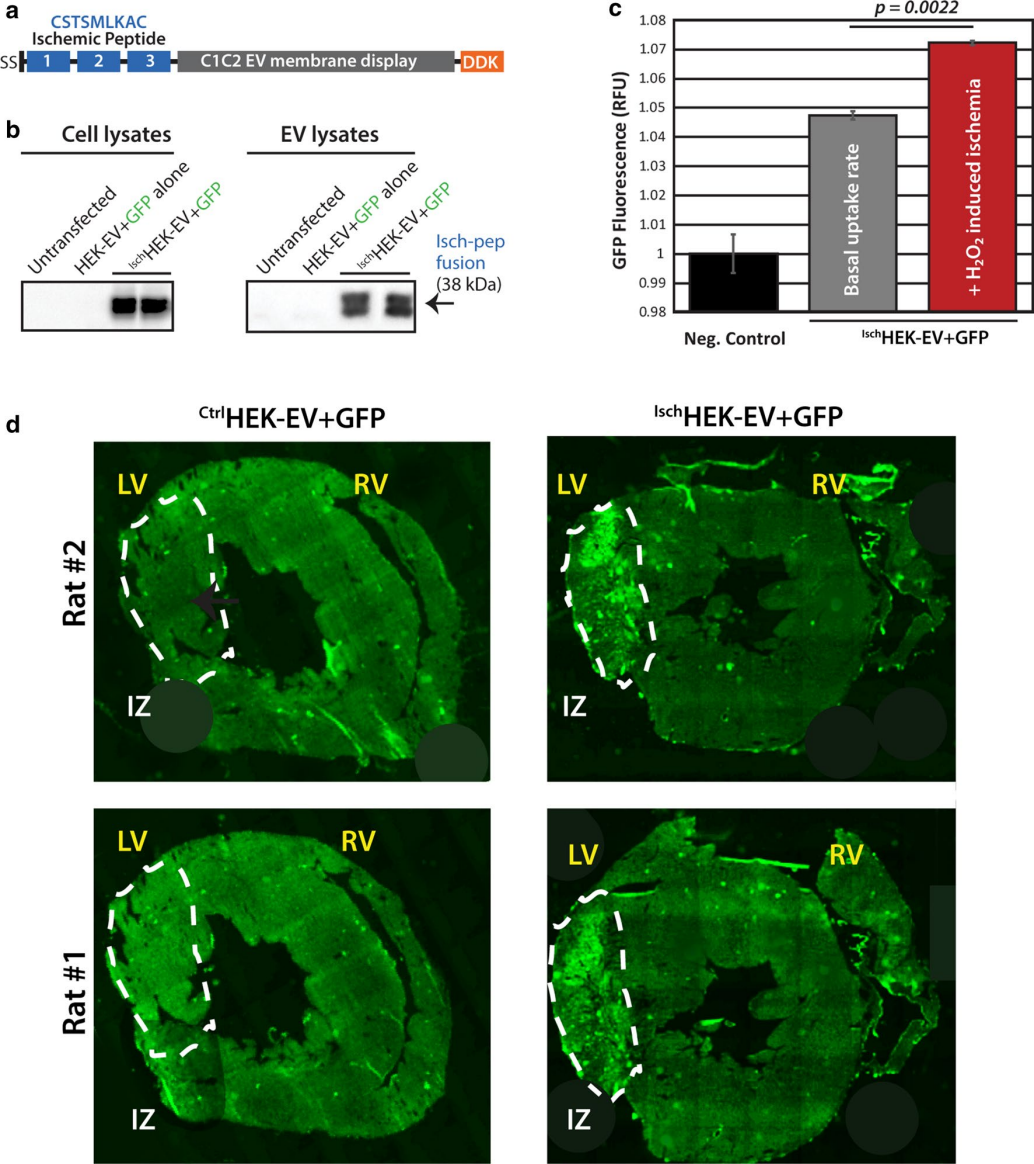
Homing of ischemic peptide HEK-EV using surface display. **a** Schematic of the lentivector expression cassette to make the fusion Ischemic peptide (Isch) coding sequence (3 repeats) fused upstream to the C1C2 domain of the human lactadherin protein (for EV membrane surface display) along with a C-terminal DDK flag tag (to detect by Western blot). **b** Western blot data confirming the expression of the fusion surface display protein in cells and on secreted exosomes. **c** Pooled FACS data of HEK-EV+GFP uptake assays with NRVM oxidative stress assays. **d** Immunofluorescent rat heart tissue section images from I/R models of myocardial infarction. Left ventricle (LV), right ventricle (RV) are labeled and ischemic zone (IZ) areas are encircled and labeled. n = 2 rats per experimental group, all rats received I/R injury

Finally, we implemented a complementary approach where transgenic parental cells are engineered to secrete EVs with targeting proteins using the lactadherin C1C2 domain for membrane surface display [29]. We tested the lactadherin surface display technique and fused three copies of the ischemia-targeting peptide sequence to make a C1C2 display fusion expression construct. This construct was used to overexpress the fusion protein and produce engineered EVs from human embryonic kidney (HEK) cells (Fig. 7a, b). To track uptake of the ^Isch^HEK-EVs, we combined a reporter loading technology [65] along with the surface display construct such that these EVs also contained GFP cargo and verified HEK-EV GFP loading by bead FACS assays (Additional file 3: Figure S3A, B). Identical experiments to those using the ischemic peptide cloaks were conducted, but this time using GFP-loaded ^Ctrl^HEK-EV or ^Isch^HEK-EV. ^Isch^HEK-EVs conferred significantly enhanced uptake (p = 0.0022) in ischemic NRVMs when compared to control, untargeted ^Ctrl^HEK-EVs (Fig. 7c). When GFP-loaded ^Isch^HEK-EVs were injected as in Fig. 6, the ischemic peptide surface display also directed the HEK-EV-mediated delivery of loaded GFP cargo to ischemic myocardial tissue similar to what was observed for the ischemic peptide cloaked EVs (Fig. 7d). Thus, our third objective in this study clearly reveals that cloaking or surface display-decorating CDC-EV with the ischemia homing peptide greatly enhances localization to injured myocardium by simple IV administration, and not just to heart tissue in general. Thus, both cloaking and transgenic surface display produce EVs with enhanced homing to ischemic tissue.

## Discussion

The field of EV-based drug delivery has greatly expanded over the past several years. Reports describing loading cargos into EVs such as siRNAs [33, 66, 67], mRNA [31], therapeutic proteins [68–70] and chemotherapeutics [71, 72] have clearly demonstrated that vesicles can function as nanocarriers. EVs have intrinsic, favorable lipid and surface protein composition that offer cellular uptake advantages over existing lipid-based delivery systems [67, 73]. EVs also show lower immunogenicity profiles [74, 75] and retain longer half-lives in circulation when compared to other vesicle-based delivery systems [76]. Loading EVs with valued cargo is one achievement; programming their delivery to specific target tissues would be a significant enhancement to such a therapeutic. The approach most thoroughly described here manipulates CDC-EVs, ex vivo, to improve tissue targeting and potentially therapeutic value. A recent report used a similar approach but with somewhat different findings. A phospholipid embedding agent, dioleoylphosphatidyl-ethanolamine *N*-hydroxysuccinimide (DOPE), was conjugated with a copy of the ischemic peptide sequence (termed CHP in that report) and added to CDC-EVs [77]. Such modified EVs homed to the heart in vivo, independent of whether or not the heart was ischemic. In contrast, we find that ischemia peptide surface display targeting and membrane cloaking both home EVs specifically to areas of damaged myocardium that had been subjected to I/R. We cannot explain the discrepancy between our findings and those of Vandergriff et al. [77], but we note that our findings are consistent with the initial panning method to isolate the ischemic homing peptide [57] and with its later use to enhance targeting of liposomes to infarcted myocardium [60].

The two EV surface engineering methods described here to target injured myocardium using the ischemic homing peptide showed equivalent results. The two methods have complementary advantages and limitations. Surface display approaches require transgenic modification of the producer cells. This can produce EVs with desired properties reproducibly, but the process is tedious in non-immortalized primary cells. To establish a cell line stably expressing the surface display fusion protein, transgenes must be introduced by viral transduction or transposon integration. Building surface display cell lines is time-consuming and expensive. Additionally, surface display techniques using antibodies can be problematic. A known, validated single-chain variable fragment sequence must be used to fuse to the C1C2 display domain and expressed in the producer cells. Cloaking lends itself to utilizing any biotinylated antibody; thousands are commercially available for testing. Adding a biotinylated fluorophore, such as FITC, PE or Qdots is a feature not available for surface display, yet is a simple addition to EVs using the cloaking platform. The cloaking technology described here, where EVs are produced ab initio from any parental cell line, is easy to implement, economical, and equally effective to surface display (based on the limited comparisons presented here; see Figs. 5, 6, and 7).

## Conclusions

We have described and implemented a molecular platform method to place targeting moieties, such as antibodies, homing peptides and other biological ligands, directly onto EV surfaces to enhance tissue targeting. The platform is simple to employ and quick: cloaking EVs requires less than 1-h hands-on experimental time. We show proof-of-concept studies utilizing three different types of cloaks (fluorescent molecules, targeting antibodies, and homing peptides) across diverse cell culture types for uptake studies, as well as animal models to verify tissue localization of engineered EVs. The system may be used to screen for top targeting molecules to direct EVs to desired destinations in cell culture models that are otherwise resistant to EV uptake, and to program EV delivery to organs and discreet tissues with animal models via delivery by IV to the circulation, thus enabling the ability to craft EVs for specific purposes.

## Methods

### Isolation of EVs

There is no single correct method for isolating extracellular vesicles and exosomes. The principal methods rely on: (1) ultracentrifugation, (2) size-exclusion (e.g., ultrafiltration and/or chromatography), (3) immunological separation (e.g., antibody-bead capture), and (4) polymer-based precipitation. Each of these methods offers tradeoffs between purity (i.e., protein-to-particle ratio, with the goal of minimizing non-EV proteins that may be present in the conditioned media), yield (number of particles) and quality (preservation of particle integrity). Purity of the EV preparation has been shown to influence potency, as large protein contaminants such as extracellular matrix proteins coat receptors necessary for endocytosis [78] and signal transduction in target cells. We favor ultrafiltration due to its convenient application, scalability, satisfactory yield, and purity.

The data presented here used EVs prepared by conditioning CDC cells for 15 days in glucose-containing serum-free basal media (which increases potency). Conditioned media was cleared of cellular debris using sterile vacuum filtration (0.45 μm filter). EVs were isolated using ultrafiltration by centrifugation (UFC) with a molecular weight cutoff of 10 kDa, which retains the bioactive fraction (Vivacell 10 kDa MWCO Filtration unit). Glucose was included in the basal media as it enhances production of vesicles and increases their resilience as manifested by less cryodamage during repeated freeze/thaw cycles. Characterization of EV preparations occurred at three levels; identity, bioactivity, and potency. Primary EV characterization methods included verifying particle size distributions proximate to previous descriptions in the literature (30–150 nm), presence of salient exosome markers including tetraspanins (CD63, CD9, and CD81), the absence of cell debris contaminants (e.g., endoplasmic reticulum proteins such as calnexin), and intactness of vesicles (e.g., RNA protection following RNAse treatment). We routinely characterize all EV batches in terms of (1) particle size, number, and concentration (by nanoparticle tracking analysis, NanoSight NS300, Malvern [79]); (2) RNA and protein content; (3) qPCR quantification of selected miRs and Y RNA fragments which are associated with CDC-EV efficacy [16, 24]; (4) response to IV-injected EVs in our standard in vivo potency assay of mouse AMI [17]; and (5) confirmation of surface ligand remodeling and presence of tetraspanins/absence of calnexin.

### EV engineering

We conjugated streptavidin (STVDN) with 1,2-bis(dimethylphosphino)ethane: polyethylene glycol 5k (DMPE-PEG) to create a modular EV membrane anchoring platform (DMPE-PEG-STVDN; abbreviated DPS) using a custom chemical synthesis service (NANOCS, Inc.). DMPE-PEG “cloak” embeds into vesicle membranes and serves as an anchor for coupling fluorescent molecules or ligand proteins [37, 80]. Thus, any biotinylated molecule (e.g., antibodies) can be coupled to the DPS anchor to decorate vesicle membranes for targeted delivery. The cloaking reaction was straightforward. First, the DPS anchor was incubated with the biotinylated molecule in a 1:5 ratio, e.g., 10 µg DPS plus 50 µg bio-FITC (NANOCS, cat# PG2-BNFC-5k) or bio-PE (Thermo Fisher Scientific, cat# P811), bio-Antibody, bio-Homing peptide, bio-Qdot 655 (Thermo Fisher Scientific, cat# Q10321MP) for 10 min at 25 °C. Next, the complex was mixed with CDC-EV (10^9^–10^11^ particles in 500 µL) and incubated for 10 min at 37 °C. The resulting suspension was concentrated by 100 kDa UFC. The flow-through (bottom of column, containing unincorporated complexes and dyes) was discarded and the retentate (top of column, containing the cloaked CDC-EV) was washed 2× with PBS by UFC. As a negative control, CDC-EV were incubated with bio-FITC or bio-PE without the DPS anchor. The same reaction ratios were employed for cardiac fibroblast targeting with α-DDR2 biotinylated rabbit polyclonal antibody (LifeSpan Biosciences, cat# LS-C255960, rabbit IgG isotype control, Abcam cat# ab200208). Muscle targeting, biotinylated peptide (H_2_N-ASSLNIAGGASSLNIAGGASSLNIA(KLC_Biot_)-OH) was synthesized by New England Peptide, Inc. and the ischemia-targeting peptide (H_2_N-CSTSMLKACGGCSTSMLKACGGCSTSMLKAC_Biot_-OH) was synthesized using ABclonal, Inc. custom peptide synthesis services. Ischemia-targeting peptide approach was further validated using transfected human embryonic kidney (HEK293) cells to produce engineered EVs with (1) a GFP lentivector that targets to secreted vesicles (XO-GFP; XPAKGFP, System Biosciences), and (2) the ischemic targeting peptide CSTSMLKAC coding sequence was cloned in triplicate and fused at the N-terminus to the C1C2 domain of the human lactadherin protein (surface display technology) [29] along with a C-terminal DDK flag tag (to detect by Western blot). HEK293 cells were transfected with XO-GFP plasmid alone or cotransfected Ischemic peptide surface display lentivector plasmid using standard Lipofectamine procedures (Invitrogen). The next day, media was exchanged to serum-free medium. Twenty-four hours later, conditioned medium was harvested, cell debris removed (3200×*g* for 20 min), and EVs isolated by UFC. When examined by nanoparticle tracking analysis (NanoSight), EVs revealed typical size (mode diameter ~ 130 nm) and concentration (10^9^ particles/mL) found with EVs and exosomes. Successful loading of XO-GFP and ischemic peptide surface display into HEK EVs (^Isch^HEK-EV) was confirmed by flow cytometry with magnetic bead capture (MagCapture™ Exosome Tim4, WAKO [81]) assays (Additional file 3: Figure S3A, B) as well as standard SDS-PAGE Western blot methods [82] using the following antibodies: anti-DDK Flag tag Rabbit polyclonal antibody Abcam cat# ab1162; anti-TurboGFP rabbit polyclonal antibody, Evrogen cat# AB513; secondary Anti-rabbit IgG, HRP-linked Antibody Cell Signaling technologies, cat# 7074S or Abcam secondary Goat Anti-Rabbit Alexa Fluor^®^ 488 (IgG H&L) cat# ab150077.

### NanoSight EV particle analysis

The NanoSight technique employs Nanoparticle Tracking Analysis (NTA), a type of light scattering technology that also utilizes particle tracking by Brownian motion, that be used for sizing nanoparticles as well as counting the number of particles present in a sample that is in a natural, aqueous environment. CDC-EVs were gently vortexed at 2.5 k for 10 s and then bath sonicated for 10 min at 33 °C to ensure adequate vesicle dispersion in the solution prior to NTA analysis. NanoSight measurements are carried out in 0.02 μm filtered PBS to remove any background particles and then visualized on an NS300 NanoSight instrument in either visible mode or fluorescent mode 532 nm laser with a 565 nm long pass filter, to detect Qdot 655 labeling, at ambient temperature. All measurements were made in quadruplicate with flow applied with an automated syringe pump between detections.

### Cell culture and animal models

Neonatal rat ventricular myocytes (NRVMs) were isolated from P2 neonatal Sprague–Dawley rats as previously described [48] (16). The cells were plated on fibronectin-coated 6-well plates at a density of 1.5 million cells/well in Dulbecco’s Modified Eagle Medium (DMEM) containing 10% Fetal Bovine Serum (Gibco) media and incubated at 37 °C, with 5% CO_2_ for 24 h. Following washing with serum free DMEM, the cells were incubated with control or engineered EVs (10^3^ EV/NRVM cell) for 4 h. The NRVMs where then processed (Tryple, Thermo Fisher Scientific) for flow cytometry on a BD FACS Canto II instrument. Flow data were analyzed using FlowJo^®^ software. In vivo experimental protocols were performed on 7- to 10-week-old female Wistar-Kyoto rats (Charles River Labs). To induce ischemia/reperfusion (IR) injury, rats were provided general anesthesia, and then a thoracotomy was performed at the 4th intercostal space to expose the heart and left anterior descending coronary artery. A 7–0 silk suture was then used to ligate the left anterior descending coronary artery, which was subsequently removed after 45 min to allow for reperfusion for 20 min [23]. PBS sham, control or targeted EVs (10^9^ particles in 1 mL PBS vehicle) were injected into test animals via slow tail vein injection. After 48 h, the animals were sacrificed and whole organ tissues collected for Qdot fluorescence biodistribution quantitation using either fluorescent plate reader (SpectraMax iD3; excitation/emission settings: 450 nm/655 nm) or tissue imaging using a Xenogen IVIS Lumina III Series instrument with Qdot 655 detection settings.

### Tissue analysis

Rat hearts were arrested in diastole after intraventricular injection of 10% KCl and excised, washed in PBS, and cut into serial slices of ~ 1 mm in thickness (from apex to basal edge of infarction). Heart tissue slices were incubated with 2,3,5-triphenyl-2*H*-tetrazolium chloride (Sigma, TTC, 1% solution in PBS) for 20 min in the dark, washed with PBS, and then imaged to identify infarcted areas from viable tissue (white versus red, respectively). The same heart tissue slices were embedded in optimum cutting temperature solution in a base mold/embedding ring block (Tissue Tek). Tissue blocks were immediately frozen by submersion in cold 2-methylbutane. Hearts were then sectioned at a thickness of 5 μm and mounted on slides. Qdot 655 localization was performed using fluorescent image scanning with Cy5.5 filter set (Cytation 5 Cell Multi-Mode Reader). GFP biodistribution from XO-GFP-loaded ^Ctrl^HEK-EV or ^Isch^HEK-EV I/R studies were detected using the anti-TurboGFP antibody and AF488 secondary antibody combination stated earlier in “Methods”. Heart tissue sections were scanned using the Cytation instrument with the FITC filter settings to image GFP localization.

### Statistical analyses

All data are presented as mean ± SEM. Student’s unpaired t test or one-way ANOVA was used for comparisons between two groups unless otherwise noted. A value of p < 0.05 was considered significant.

## Additional files

**Additional file 1: Figure S1.** Nanoparticle tracking analysis of cloaked CDC-EV. NanoSight NTA particle tracking data profiles in visible mode for naïve CDC-EV (black) or CDC-EV plus FITC cloaks (green). Schematic representation of the CDC-EV particles are shown as circle diagrams and the particle size means and modes are indicated. n = 3 wells per NRVM experimental group; n = 4 NTA measurements per exosome experimental group.

**Additional file 2: Figure S2.** Nanoparticle tracking analysis of CDC-EV with MTP and Qdot 655 cloaks. **A.** NanoSight particle tracking sample video images of CDC-EV + Qdot 655 cloaks during data collection in either visible or fluorescent mode as indicated. **B.** Graphical representation of NanoSight NTA quantitative analyses of Qdot 655 cloak controls s after purification using 100 kDa post-reaction spin column chromatography. NanoSight profiles of control CDC-EV with Qdot 655 cloak (**C**) or Qdot 655 + MTP homing peptide cloaks (**D**). n = 4 NTA measurements per experimental group.

**Additional file 3: Figure S3.** FACS bead Tim4 assays with GFP-loaded HEK-EVs. **A.** Schematic representation of how Tim4-coupled magnetic bead FACS assays work to detect internal, loaded GFP as well as surface CD81 markers. **B.** FACS histograms of GFP-loaded HEK-EVs on Tim4 beads for GFP detection (upper panels) and for CD81 as EV positive controls (lower panels) for ^Ctrl^HEK-EV or ^Isch^HEK-EV loaded with GFP.

## Abbreviations

EV: extracellular vesicle
DMPE: 1,2-bis(dimethylphosphino)ethane
PEG: polyethylene glycol
STVDN: streptavidin
bio: biotin
AF488: AlexaFluor^®^-488
HEK: human embryonic kidney
NRVM: neonatal rat ventricular myocytes
CDC: cardiosphere-derived cardiomyocytes
FITC: fluorescein isothiocyanate
PE: phycoerythrin
GFP: green fluorescent protein
Cy5: cyanine 5 far-red fluorescent dye
DOPE: dioleoylphosphatidyl-ethanolamine *N*-hydroxysuccinimide
CHP: cardiac homing peptide
PBS: phosphate buffered saline
Ctrl: control
Isch: ischemia homing peptide
Qdot: quantum dot nanocrystal
RNAse: ribonuclease
qPCR: quantitative polymerase chain reaction
I/R: ischemia/reperfusion
NTA: NanoSight tracking analysis
SEM: standard error of the mean
ANOVA: analysis of variance
kDa: kilodalton
RVG: rabies virus glycopeptide
BACE1: beta-secretase 1
UFC: ultrafiltration by centrifugation
MWCO: molecular weight cut-off
Tim4: T-cell immunoglobulin- and mucin-domain-containing molecule
FACS: fluorescent-activated cell sorting
DDR2: discoidin domain receptor tyrosine kinase 2
IgG: immunoglobulin G
HER2: human epidermal growth factor receptor 2
siRNA: small interfering ribonucleic acid
miRNA or miR: micro ribonucleic acid
mRNA: messenger ribonucleic acid
TTC: 2,3,5-triphenyl-2*H*-tetrazolium chloride
CEA: carcinoembryonic antigen
AMI: acute myocardial infarction
HRP: horseradish peroxidase
DMEM: Dulbecco’s Modified Eagle Medium

## References

[1] Atkin-Smith GK (2017). Isolation of cell type-specific apoptotic bodies by fluorescence-activated cell sorting. Sci Rep.

[2] Panagiotou N (2016). Microvesicles as vehicles for tissue regeneration: changing of the guards. Curr Pathobiol Rep.

[3] Raposo G, Stoorvogel W (2013). Extracellular vesicles: exosomes, microvesicles, and friends. J Cell Biol.

[4] Dragovic RA (2011). Sizing and phenotyping of cellular vesicles using nanoparticle tracking analysis. Nanomedicine.

[5] Andreu Z, Yanez-Mo M (2014). Tetraspanins in extracellular vesicle formation and function. Front Immunol.

[6] Navakanitworakul R (2016). Characterization and small RNA content of extracellular vesicles in follicular fluid of developing bovine antral follicles. Sci Rep.

[7] Zhang Y, Wang XF (2015). A niche role for cancer exosomes in metastasis. Nat Cell Biol.

[8] Steinbichler TB (2017). The role of exosomes in cancer metastasis. Semin Cancer Biol.

[9] McGough IJ, Vincent JP (2016). Exosomes in developmental signalling. Development.

[10] Qu P (2017). Effects of embryo-derived exosomes on the development of bovine cloned embryos. PLoS ONE.

[11] Greening DW (2015). Exosomes and their roles in immune regulation and cancer. Semin Cell Dev Biol.

[12] Ellwanger JH (2016). Exosomes are possibly used as a tool of immune regulation during the dendritic cell-based immune therapy against HIV-I. Med Hypotheses.

[13] Ferreira ADF (2017). Extracellular vesicles from adipose-derived mesenchymal stem/stromal cells accelerate migration and activate AKT pathway in human keratinocytes and fibroblasts independently of mir-205 activity. Stem Cells Int.

[14] Golchin A, Hosseinzadeh S, Ardeshirylajimi A (2018). The exosomes released from different cell types and their effects in wound healing. J Cell Biochem.

[15] Aminzadeh MA (2018). Exosome-mediated benefits of cell therapy in mouse and human models of duchenne muscular dystrophy. Stem Cell Reports.

[16] Ibrahim AG, Cheng K, Marbán E (2014). Exosomes as critical agents of cardiac regeneration triggered by cell therapy. Stem Cell Reports.

[17] Ibrahim A, Marban E (2016). Exosomes: fundamental biology and roles in cardiovascular physiology. Annu Rev Physiol.

[18] Davis DR, Zhang Y, Smith RR, Cheng K, Terrovitis J, Malliaras K, Li TS, White A, Makkar R, Marban E (2009). Validation of the cardiosphere method to culture cardiac progenitor cells from myocardial tissue. PLoS ONE.

[19] Marbán E (2018). A mechanistic roadmap for the clinical application of cardiac cell therapies. Nat Biomed Eng..

[20] Malliaras K, Li T, Luthringer D, Terrovitis J, Cheng K, Chakravarty T, Galang G, Zhang Y, Schoenhoff F, Van Eyk J, Marbán L, Marbán E (2012). Safety and efficacy of allogeneic cell therapy in infarcted rats transplanted with mismatched cardiosphere-derived cells. Circulation.

[21] Chimenti I, Smith R, Li TS, Gerstenblith G, Messina E, Giacomello A, Marbán E (2010). Relative roles of direct regeneration versus paracrine effects of human cardiosphere-derived cells transplanted into infarcted mice. Circ Res.

[22] Luther K, McGuinness M, Haar L, Xu H, Chen J, Medvedovic M, Jones WK (2016). MSC exosomes deliver cardioprotective miR-21. FASEB J.

[23] de Couto G (2017). Exosomal microRNA transfer into macrophages mediates cellular postconditioning. Circulation.

[24] Cambier L (2017). Y RNA fragment in extracellular vesicles confers cardioprotection via modulation of IL-10 expression and secretion. EMBO Mol Med.

[25] Tseliou E, Fouad J, Reich H, Slipczuk L, de Couto G, Aminzadeh M, Middleton R, Valle J, Weixin L, Marbán E (2015). *Fibroblasts rendered antifibrotic, antiapoptotic, and angiogenic by priming with cardiosphere*-*derived extracellular membrane vesicles*. J Am Coll Cardiol.

[26] de Couto G (2015). *Macrophages mediate cardioprotective cellular postconditioning in acute myocardial infarction*. J Clin Invest.

[27] Gallet R, Dawkins J, Valle J, Simsolo E, de Couto G, Middleton R, Tseliou E, Luthringer D, Kreke M, Smith RR, Marbán L (2017). Exosomes secreted by cardiosphere-derived cells reduce scarring, attenuate adverse remodelling, and improve function in acute and chronic porcine myocardial infarction. Eur Heart J.

[28] Conlan RS, Pisano S, Oliveira MI, Ferrari M, Mendes Pinto I (2017). Exosomes as reconfigurable therapeutic systems. Trends Mol Med.

[29] Delcayre A (2005). Exosome display technology: applications to the development of new diagnostics and therapeutics. Blood Cells Mol Dis.

[30] Hartman ZC (2011). Increasing vaccine potency through exosome antigen targeting. Vaccine.

[31] Wang JH, Forterre AV, Zhao J, Frimannsson DO, Delcayre A, Antes TJ, Efron B, Jeffrey SS, Pegram MD, Matin AC (2018). Anti-HER2 scFv-directed extracellular vesicle-mediated mRNA-based gene delivery inhibits growth of HER2-positive human breast tumor xenografts by prodrug activation. Mol Cancer Ther..

[32] Meyer C (2017). Pseudotyping exosomes for enhanced protein delivery in mammalian cells. Int J Nanomed.

[33] Alvarez-Erviti L (2011). Delivery of siRNA to the mouse brain by systemic injection of targeted exosomes. Nat Biotechnol.

[34] Kao SC (2004). BACE1 suppression by RNA interference in primary cortical neurons. J Biol Chem.

[35] Nishitomi K (2006). BACE1 inhibition reduces endogenous Abeta and alters APP processing in wild-type mice. J Neurochem.

[36] Sato YT (2016). Engineering hybrid exosomes by membrane fusion with liposomes. Sci Rep.

[37] Nag OK, Awasthi V (2013). Surface engineering of liposomes for stealth behavior. Pharmaceutics.

[38] Won YW, Patel AN, Bull DA (2014). Cell surface engineering to enhance mesenchymal stem cell migration toward an SDF-1 gradient. Biomaterials.

[39] Zeringer E (2015). Strategies for isolation of exosomes. Cold Spring Harb Protoc.

[40] Li P (2017). Progress in exosome isolation techniques. Theranostics.

[41] Ailawadi S (2015). Pathologic function and therapeutic potential of exosomes in cardiovascular disease. Biochim Biophys Acta.

[42] van Amerongen MJ, Engel FB (2008). Features of cardiomyocyte proliferation and its potential for cardiac regeneration. J Cell Mol Med.

[43] Fredj S (2005). Interactions between cardiac cells enhance cardiomyocyte hypertrophy and increase fibroblast proliferation. J Cell Physiol.

[44] Zeisberg EM, Kalluri R (2010). Origins of cardiac fibroblasts. Circ Res.

[45] LaFramboise WA (2007). Cardiac fibroblasts influence cardiomyocyte phenotype in vitro. Am J Physiol Cell Physiol.

[46] Smiley D (2014). Increased fibrosis and progression to heart failure in MRL mice following ischemia/reperfusion injury. Cardiovasc Pathol.

[47] Olaso E (2002). Discoidin domain receptor 2 regulates fibroblast proliferation and migration through the extracellular matrix in association with transcriptional activation of matrix metalloproteinase-2. J Biol Chem.

[48] Golden HB (2012). Isolation of cardiac myocytes and fibroblasts from neonatal rat pups. Methods Mol Biol.

[49] Hyvonen M, Laakkonen P (2015). Identification and characterization of homing peptides using in vivo peptide phage display. Methods Mol Biol.

[50] Pasqualini R, Ruoslahti E (1996). Organ targeting in vivo using phage display peptide libraries. Nature.

[51] Rajotte D, Ruoslahti E (1999). Membrane dipeptidase is the receptor for a lung-targeting peptide identified by in vivo phage display. J Biol Chem.

[52] Li J, Feng L, Jiang X (2015). In vivo phage display screen for peptide sequences that cross the blood–cerebrospinal-fluid barrier. Amino Acids.

[53] Li J (2012). Identification of peptide sequences that target to the brain using in vivo phage display. Amino Acids.

[54] Denby L (2007). Development of renal-targeted vectors through combined in vivo phage display and capsid engineering of adenoviral fibers from serotype 19p. Mol Ther.

[55] Ghosh D, Barry MA (2005). Selection of muscle-binding peptides from context-specific peptide-presenting phage libraries for adenoviral vector targeting. J Virol.

[56] Samoylova TI, Smith BF (1999). Elucidation of muscle-binding peptides by phage display screening. Muscle Nerve.

[57] Kanki S (2011). Identification of targeting peptides for ischemic myocardium by in vivo phage display. J Mol Cell Cardiol.

[58] Morgan JE (1994). Myogenic cell lines derived from transgenic mice carrying a thermolabile T antigen: a model system for the derivation of tissue-specific and mutation-specific cell lines. Dev Biol.

[59] Muses S, Morgan JE, Wells DJ (2011). A new extensively characterised conditionally immortal muscle cell-line for investigating therapeutic strategies in muscular dystrophies. PLoS ONE.

[60] Won YW (2013). Targeted gene delivery to ischemic myocardium by homing peptide-guided polymeric carrier. Mol Pharm.

[61] Janero DR, Hreniuk D, Sharif HM (1993). Hydrogen peroxide-induced oxidative stress to the mammalian heart–muscle cell (cardiomyocyte): nonperoxidative purine and pyrimidine nucleotide depletion. J Cell Physiol.

[62] Michalet X (2005). Quantum dots for live cells, in vivo imaging, and diagnostics. Science.

[63] Courty S, Dahan M (2013). Ultrasensitive imaging in live cells using fluorescent quantum dots. Cold Spring Harb Protoc.

[64] Pic E (2010). Fluorescence imaging and whole-body biodistribution of near-infrared-emitting quantum dots after subcutaneous injection for regional lymph node mapping in mice. Mol Imaging Biol.

[65] Shen B (2011). Protein targeting to exosomes/microvesicles by plasma membrane anchors. J Biol Chem.

[66] Ha D, Yang N, Nadithe V (2016). Exosomes as therapeutic drug carriers and delivery vehicles across biological membranes: current perspectives and future challenges. Acta Pharm Sin B.

[67] Shahabipour F, Banach M, Sahebkar A (2016). Exosomes as nanocarriers for siRNA delivery: paradigms and challenges. Arch Med Sci.

[68] Sterzenbach U (2017). Engineered exosomes as vehicles for biologically active proteins. Mol Ther.

[69] Yim N (2016). Exosome engineering for efficient intracellular delivery of soluble proteins using optically reversible protein–protein interaction module. Nat Commun.

[70] Haney MJ (2015). Exosomes as drug delivery vehicles for Parkinson’s disease therapy. J Control Release.

[71] Mahaweni NM (2013). Tumour-derived exosomes as antigen delivery carriers in dendritic cell-based immunotherapy for malignant mesothelioma. J Extracell Vesicles.

[72] Vader P (2016). Extracellular vesicles for drug delivery. Adv Drug Deliv Rev.

[73] Conlan RS (2017). Exosomes as reconfigurable therapeutic systems. Trends Mol Med.

[74] Zhu X (2017). Comprehensive toxicity and immunogenicity studies reveal minimal effects in mice following sustained dosing of extracellular vesicles derived from HEK293T cells. J Extracell Vesicles.

[75] Lakhal S, Wood MJ (2011). Exosome nanotechnology: an emerging paradigm shift in drug delivery: exploitation of exosome nanovesicles for systemic in vivo delivery of RNAi heralds new horizons for drug delivery across biological barriers. BioEssays.

[76] Turturici G (2014). Extracellular membrane vesicles as a mechanism of cell-to-cell communication: advantages and disadvantages. Am J Physiol Cell Physiol.

[77] Vandergriff A (2018). Targeting regenerative exosomes to myocardial infarction using cardiac homing peptide. Theranostics.

[78] Paolini L (2016). Residual matrix from different separation techniques impacts exosome biological activity. Sci Rep.

[79] Filipe V, Hawe A, Jiskoot W (2010). Critical evaluation of nanoparticle tracking analysis (NTA) by NanoSight for the measurement of nanoparticles and protein aggregates. Pharm Res.

[80] Won YW, Patel AN, Bull DA (2014). Cell surface engineering to enhance mesenchymal stem cell migration toward an SDF-1 gradient. Biomaterials.

[81] Nakai W (2016). A novel affinity-based method for the isolation of highly purified extracellular vesicles. Sci Rep.

[82] Blancher C, Jones A (2001). SDS-PAGE and Western blotting techniques. Methods Mol Med.

