# Supplementary figures and images for: Targeting extracellular vesicles to injured tissue using membrane cloaking and surface display
Source: J Nanobiotechnology. 2018 Aug 30;16:61. doi: 10.1186/s12951-018-0388-4 (PMC6116387; doi:10.1186/s12951-018-0388-4)

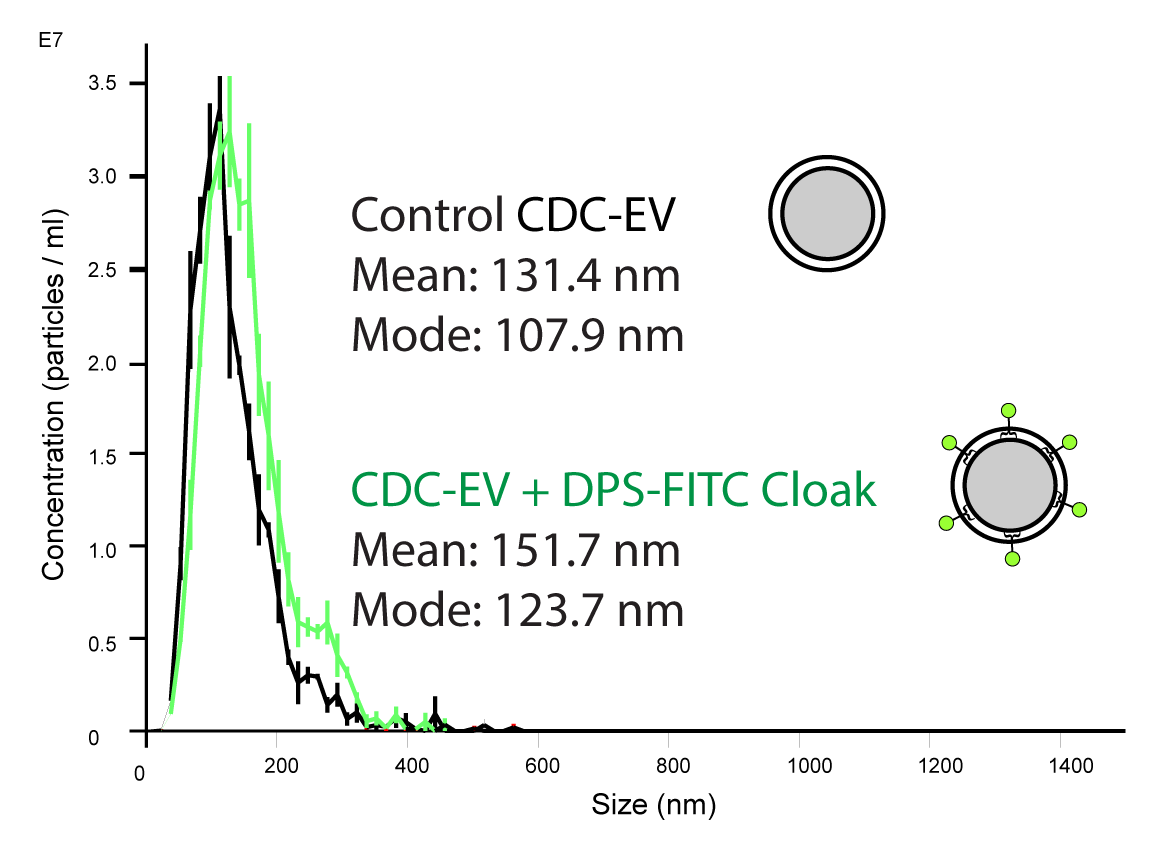

Supplement: Supplementary file 1 — Additional file 1: Figure S1. Nanoparticle tracking analysis of cloaked CDC-EV. NanoSight NTA particle tracking data profiles in visible mode for naïve CDC-EV (black) or CDC-EV plus FITC cloaks (green). Schematic representation of the CDC-EV particles are shown as circle diagrams and the particle size means and modes are indicated. n = 3 wells per NRVM experimental group; n = 4 NTA measurements per exosome experimental group. [file 12951_2018_388_MOESM1_ESM.tif]

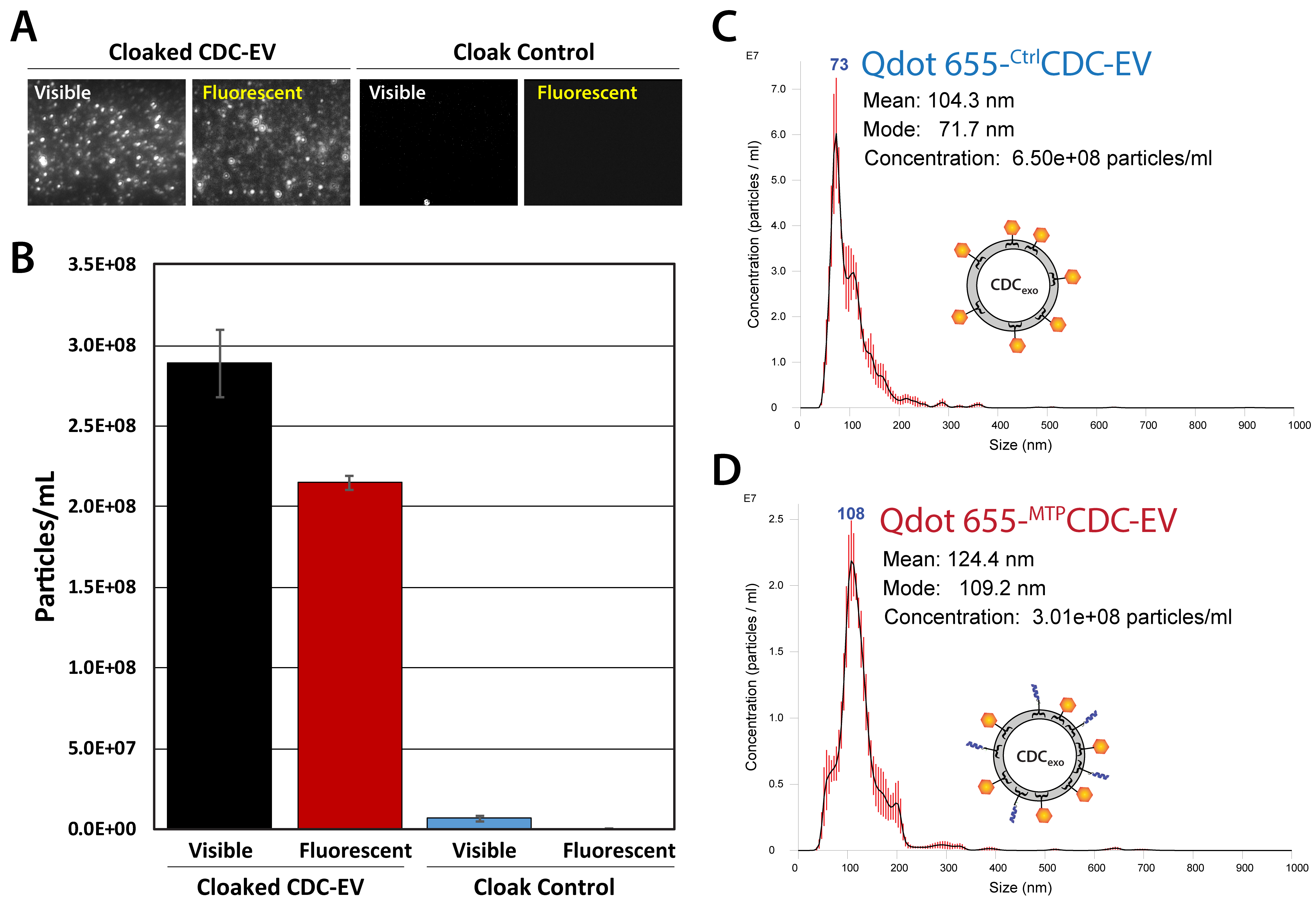

Supplement: Supplementary file 2 — Additional file 2: Figure S2. Nanoparticle tracking analysis of CDC-EV with MTP and Qdot 655 cloaks. A. NanoSight particle tracking sample video images of CDC-EV + Qdot 655 cloaks during data collection in either visible or fluorescent mode as indicated. B. Graphical representation of NanoSight NTA quantitative analyses of Qdot 655 cloak controls s after purification using 100 kDa post-reaction spin column chromatography. NanoSight profiles of control CDC-EV with Qdot 655 cloak (C) or Qdot 655 + MTP homing peptide cloaks (D). n = 4 NTA measurements per experimental group. [file 12951_2018_388_MOESM2_ESM.tif]

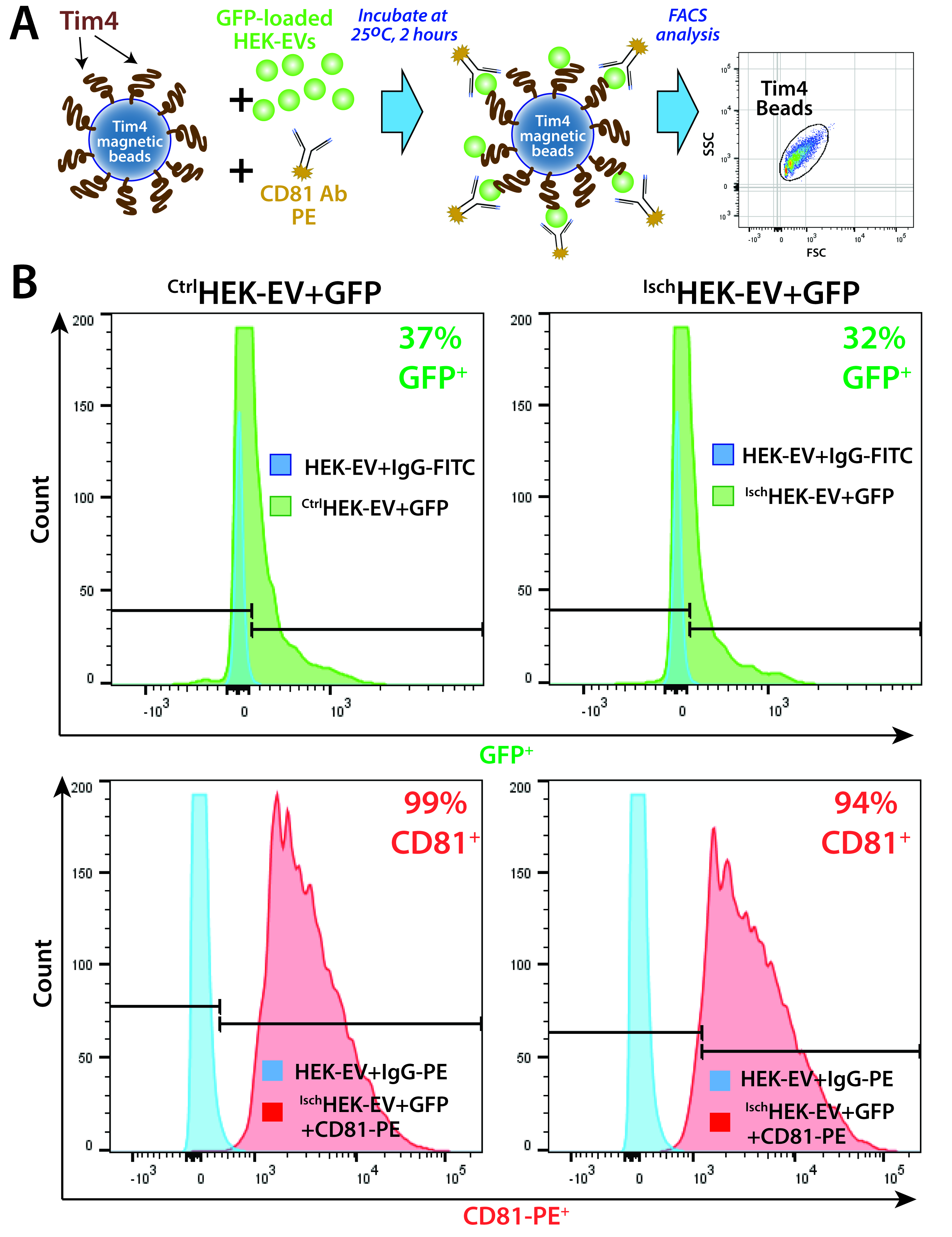

Supplement: Supplementary file 3 — Additional file 3: Figure S3. FACS bead Tim4 assays with GFP-loaded HEK-EVs. A. Schematic representation of how Tim4-coupled magnetic bead FACS assays work to detect internal, loaded GFP as well as surface CD81 markers. B. FACS histograms of GFP-loaded HEK-EVs on Tim4 beads for GFP detection (upper panels) and for CD81 as EV positive controls (lower panels) for CtrlHEK-EV or IschHEK-EV loaded with GFP. [file 12951_2018_388_MOESM3_ESM.tif]
